# Speech Intelligibility During Clinical and Low Frequency

**DOI:** 10.3390/brainsci10010026

**Published:** 2020-01-02

**Authors:** John J. Sidtis, Diana Van Lancker Sidtis, Ritesh Ramdhani, Michele Tagliati

**Affiliations:** 1 Brain and Behavior Laboratory, Geriatrics Division, the Nathan Kline Institute, 140 Old Orangeberg Road, Building 35, Orangeburg, NY 10962, USA; diana.sidtis@nyu.edu; 2 Department of Psychiatry, New York University-Langone School of Medicine, 550 First Avenue, New York, NY 10016, USA; 3 Department of Communicative Sciences and Disorders, New York University Steinhardt School, 665 Broadway, 9th Floor, New York, NY 10012, USA; 4 Department of Neurology, Donald and Barbara Zucker School of Medicine at Hofstra/Northwell, 500 Hofstra University, Hempstead, NY 11549, USA; ritesh.a.ramdhani@gmail.com; 5 Department of Neurology, Cedars-Sinai Medical Center, 127 S San Vicente Blvd, AHSP A6318, Los Angeles, CA 90048, USA; michele.tagliati@cshs.org

**Keywords:** speech, Parkinson’s disease, deep brain stimulation, voice, articulation

## Abstract

Deep brain stimulation (DBS) of the subthalamic nucleus (STN) has become an effective and widely used tool in the treatment of Parkinson’s disease (PD). STN-DBS has varied effects on speech. Clinical speech ratings suggest worsening following STN-DBS, but quantitative intelligibility, perceptual, and acoustic studies have produced mixed and inconsistent results. Improvements in phonation and declines in articulation have frequently been reported during different speech tasks under different stimulation conditions. Questions remain about preferred STN-DBS stimulation settings. Seven right-handed, native speakers of English with PD treated with bilateral STN-DBS were studied off medication at three stimulation conditions: stimulators off, 60 Hz (low frequency stimulation—LFS), and the typical clinical setting of 185 Hz (High frequency—HFS). Spontaneous speech was recorded in each condition and excerpts were prepared for transcription (intelligibility) and difficulty judgements. Separate excerpts were prepared for listeners to rate abnormalities in voice, articulation, fluency, and rate. Intelligibility for spontaneous speech was reduced at both HFS and LFS when compared to STN-DBS off. On the average, speech produced at HFS was more intelligible than that produced at LFS, but HFS made the intelligibility task (transcription) subjectively more difficult. Both voice quality and articulation were judged to be more abnormal with DBS on. STN-DBS reduced the intelligibility of spontaneous speech at both LFS and HFS but lowering the frequency did not improve intelligibility. Voice quality ratings with STN-DBS were correlated with the ratings made without stimulation. This was not true for articulation ratings. STN-DBS exacerbated existing voice problems and may have introduced new articulatory abnormalities. The results from individual DBS subjects showed both improved and reduced intelligibility varied as a function of DBS, with perceived changes in voice appearing to be more reflective of intelligibility than perceived changes in articulation.

## 1. Introduction

High frequency, chronic stimulation of the subthalamic nuclei (STN) has become a widespread tool in the treatment of levodopa responsive Parkinson’s disease (PD), minimizing tremor and bradykinesia. Similar to levodopa, however, deep brain stimulation (DBS) of the STN has had a less impressive impact on the axial symptoms, including balance, gait, and speech. It has been suggested that stimulating the STN at lower frequencies (LFS) may have therapeutic effects comparable to that of high frequency stimulation (HFS) with some additive benefits for these otherwise untreated behaviors. Some studies focused on gait to evaluate the effects of different STN frequencies, while others have examined speech under these conditions. This paper begins with a brief review of gait studies to further understand of the effects of STN frequency settings, followed by a consideration of selected studies of speech. Both behaviors, gait and speech, have been shown to yield variable results with this form of therapy.

Moreau et al. [1] found less freezing of gait and better completion time in a gait task at LFS compared to HFS. Xie et al. [2] reported that LFS reduced freezing of gait in two subjects who developed this condition with HFS stimulation. A larger sample also found that the LFS reduced the frequency of aspiration and freezing of gait [3]. Ricchi et al. [4] studied gait in subjects who were reduced to LFS from HFS. Over a follow-up period, three were returned to HFS, five showed improvement and three showed no change. Brozova et al. [5] found that three of 12 subjects could not tolerate LFS, but the remaining subjects had improvements in gait, balance, and speech. Khoo et al. [6] found that LFS had a superior effect on the Unified Parkinson’s Disease Related Scale (UPDRS) motor score compared to HFS. 

In contrast, Phibbs et al. [7] compared LFS and HFS of the STN and found no difference in stride length in gait. Similarly, Vallabhajosula et al. [8] found that UPDRS rating, step length, and gait speed improved with both HFS and LFS, but found no difference between the stimulation frequencies. Sidiropoulos et al. [9] found no significant improvements in gait, balance, or speech with LFS. While LFS may not be superior to HFS in all situations, Ramdhani et al. [10] reported that in subjects who did not respond positively to HFS, gait was improved with 60 Hz stimulation. These results were reviewed by Baizabal-Carvallo and Alonso-Juarez [11].

Analogous to gait, studies of the effects of STN-DBS on speech have obtained mixed results. It is generally believed that STN-DBS has adverse effects on speech [12,13], but the exact nature and extent of these changes have been elusive. Studies have typically evaluated one or more speech tasks including sustained vowel productions, syllable repetition, reading or repetition of text, or in some cases, spontaneously spoken monologues. Spoken material has been collected on or off medication, in subjects with varying degrees of Parkinsonian dysarthria. 

Dromey et al. [14] recorded monologue speech and sustained phonation examining the effects of STN-DBS. Small, but significant increases in the vocal intensity and fundamental frequency variability were observed during monologue speech with medication and STN-DBS on. However, it was concluded that “the overall impact is not substantial and would not represent a functionally useful change in speech performance” (p. 1136). Changes in sustained vowel performance were not reported. Similarly, Sidtis et al. [15] found that harmonic-to-noise ratio (HNR), low in the conversation task, increased with STN-DBS during conversation to the level measured for repetition. Gentile et al. [16] recorded sustained vowels and repeated nonsense words, real words, phrases, and sentences with STN-DBS on and off. Shorter maximum sustained phonation times for one of the two vowels tested and longer durations for one of the two nonsense syllables tested were reported with STN-DBS off for males but not females. Klostermann et al. [17] evaluated sustained vowel productions and reading from a standard passage. The subjective ratings of speech performance by the subjects and their clinicians suggested poorer speech with STN-DBS on compared to STN-DBS off. Acoustic analyses of the sustained vowels revealed no differences between STN-DBS on and off. Maximum phonation times did increase with STN-DBS on as did the speech rate during the reading passage. Additional varied studies comparing STN-DBS on and off describe a similar array of mixed results on speech [18,19].

The effects of varying the parameters of STN-DBS on speech have also been studied. Törnquist et al. [20] increased or decreased the amplitude of STN-DBS by 25% and used frequencies of 70 (LFS), 130 (HFS), or 185 Hz (HFS) while subjects read a standard text and five nonsense sentences. Eleven different STN-DBS settings were evaluated with setting conditions that were separated by 3 to 5 min. Each setting was in effect for 3 min for speech evaluation. Speech recordings were evaluated by five native speakers and five professional speech therapists who provided transcriptions and subjective ratings of speech and voice characteristics. At the normally used clinical settings, there were no significant differences in speech performance comparing STN-DBS on and off. Increasing the amplitude of STN-DBS reduced the number of correctly transcribed words and increased overall rated intelligibility and articulation problems. The number of transcribed words was greater and the rated articulation showed fewer abnormalities with LFS. 

Tripoliti et al. [21] evaluated low voltage (2v), high voltage (4v), inside the STN, outside of the STN, at the normal clinical settings, and with the stimulators off. Subjects were tested off medication producing a sustained vowel, reading passages from a standard intelligibility test, and producing a one minute monologue. At the normal clinical settings, intelligibility scores did not differ comparing STN-DBS on and off but high voltage significantly reduced intelligibility. Speech intensity was not significantly affected by voltage changes for any of the speech tasks. 

The effects of high and low frequency STN-DBS on speech were also evaluated in several studies. The effects of LFS (60 Hz) and HFS (130) on the production of the sustained vowel /*a*/, repeating the syllable /*pa*/, producing a forced expired volume, and the speech rating on the UPDRS were examined [22]. There was a marginal difference on the UPDRS speech rating, with slightly less impairment with LFS compared to HFS. The only significant difference in the acoustic measures was an increase in F0 during LFS compared to HFS for the female speakers. 

Grover et al. [23] evaluated speech during LFS (60 Hz and 80 Hz) and HFS (110 Hz, 130 HZ, and 200 Hz). Voltage was increased or decreased as a function of frequency to maintain constant total electrical energy delivered. Subjects were assessed off medication and speech was assessed with a standardized reading test of intelligibility and a 60 s monologue. Performance on a standard intelligibility test decreased as STN-DBS frequency increased, but pair-wise comparisons between individual frequencies were not significant. Using a different scoring method with the same test material, there was a similar overall effect across frequencies with the intelligibility at 60 Hz better than intelligibility at 200 Hz. The same frequency effects were observed for the monologue speech. 

Da Cruz Morello et al. [24] compared LFS (60 Hz) to HFS (130 Hz) effects on speech using a sustained vowel and passages from a dysarthria protocol. Subjects were on medication. Three judges rated the characteristics of the speech in each condition. LFS was associated with increased weakness and instability and HFS was associated with improved phonation and articulation; the off condition was not assessed. Acoustic measures revealed no significant differences. 

In summary, across studies, the effects of STN-DBS have shown several tendencies: speech was often judged as more impaired using the UPDRS speech rating; moreover, in various speech tasks, articulation competence tended to be diminished while some aspects of phonation tended to be improved. However, these results were not always consistent across speech tasks.

The present study examined the effects of three DBS frequencies using three measures of a single speech task considered most sensitive to the PD disorder and STN-DBS, spontaneous speech. Measures were the intelligibility of speech, subjective judgements about the difficulty the intelligibility task, and subjective judgements about several characteristics of the spontaneous speech. The three conditions were STN-DBS at each subject’s clinical setting (HFS), STN-DBS at 60 Hz. (LFS), and STN-DBS off. All conditions were assessed while subjects were abstaining from Levodopa. The use of spontaneous speech is significant, as studies have shown that the effects of STN-DBS on speech are more pronounced during spontaneous speech compared to reading or repetition [25,26,27,28]. 

## 2. Methods

### 2.1. Participants

There were two groups of participants in this study: STN-DBS participants and normal listeners. Speech samples were provided by seven right-handed, native speakers of American English with Parkinson’s disease (PD) who were treated with bilateral deep brain stimulation (DBS) of the subthalamic nuclei (STN). There were six males and one female with an average age of 60.1 yrs. Speech samples were obtained at three stimulus settings: HFS, LFS, and off. The STN-DBS frequencies and other demographic characteristics for each subject are presented in Table 1.

The second group of participants consisted of 15 normal adults serving as listeners, who provided, first, the intelligibility scores and difficulty ratings for the intelligibility task, and, second, subjective ratings for abnormalities in voice, articulation, fluency, and rate. All listeners were speech pathology students in their second year of training.

### 2.2. Procedure for Obtaining Speech Samples

STN-DBS participants provided informed consent from the Mount Sinai School of Medicine for the neurological evaluation and from the Nathan Kline Institute for the speech evaluations in accordance with the Code of Ethics of the World Medical Association.

STN-DBS participants arrived at the movement disorders clinic in the morning having abstained from their medication for PD after their last dose in the previous evening. The order of the DBS stimulation conditions (HFS, LFS, off) was varied across subjects. Each of the speech evaluations lasted about 20 min. Following each speech evaluation, the DBS stimulation condition was changed and testing was repeated after a 20 min acclimation interval. Following the third evaluation, medication was resumed and DBS was returned to the individual’s clinical settings. 

### 2.3. Stimulus Development

Stimuli for this study (listening samples) were extracted from a task requesting that the subject engage in a five-minute monologue about a topic of their choice. Study participants were encouraged to choose different topics for each of these monologues. New monologues were elicited at each of the three evaluations (HFS, LFS, off), which were digitally recorded (Marantz PMD660, Mahwah NJ, USA, www.us.marantz.com) using a Shure SM10A (Chicago, IL, USA, www.shure.com) head-worn microphone. The speech samples were transcribed by two students in speech-language pathology programs and a final transcription was reviewed by a third listener. Discrepancies in the transcriptions were resolved by one of the authors (DVS). The transcribers were blind with respect to the DBS condition.

For the intelligibility portion of the study, stimuli consisted of recordings of two utterances excerpted from the monologues produced during each DBS setting for each subject. Excerpts were extracted randomly across the discourse sets. The set of excerpted recordings were played to listeners during the listening task. Excerpts prepared for the listening study were 5–10 words in length, with 1–5 syllables per word without proper nouns, abbreviations, or acronyms. All selected phrases were agreed upon by the investigators. Utterances differed across conditions, eliminating the possibility of order or practice effects. Difficulty ratings provide additional information about the experience of the listener in the process of determining the intelligibility of the spoken utterances. Although subjective, they may reflect interpretable differences across the study conditions. 

For the portion of the study requiring listeners to rate the characteristics of the speakers’ speech, a second set of utterances was excerpted from the monologues. These utterances were 10–15 s in length and did not include any of the phrases used as stimuli in the intelligibility task. Again, stimuli could not contain proper nouns, abbreviations, or acronyms and all selected phrases were agreed upon as representative of each PD-DBS speaker by two investigators. The presence or absence of speech abnormalities was not a criterion for selection. This listening task was used to obtain subjective ratings of the PD-DBS speaker’s voice quality, articulation, fluency, and rate. The ratings for each of these parameters ranged from 1 (normal) to 5 (abnormal).

### 2.4. Listener’s Tasks

The study was divided into two phases: (1) intelligibility as measured by transcription accuracy and listeners ratings of the difficulty of each transcription; and (2) subjective ratings of specific speech characteristics. During the intelligibility phase, listeners were asked to listen to the phrases, which were 5–10 words in length as described above, and write down what they heard using pen or pencil on a numbered answer sheet provided. For each item, they were also asked to rate the level of difficulty they experienced in transcribing the utterance using a scale of 1 (easy) to 5 (difficult) and circling one of these numbers on the answer sheet for each item. No linguistic support was provided for the transcriptions; answer sheets consisted only of a number and a blank line for each stimulus item.

During second phase (the subjective ratings of speech characteristics), the same listeners who provided transcriptions and difficulty ratings listened to a different set of stimuli (10–15 seconds in length) and rated the stimuli using five-point scales on four parameters: voice, articulation, fluency, and rate of speech. Ratings on each scale ranged from 1 (normal) to 5 (abnormal). Listeners were asked to complete the task either individually or in small groups of 2–4 people. The stimuli were played on a Marantz Professional CD Recorder (CDR300) using a ROLLS Headphone Amplifier (RA53b) (Murray, UT, USA, www.rolls.com) with five individually controlled outputs and SONY MDR 7502 professional headphones (Tokyo, Japan, www.sony.net). The listeners were asked to individually determine their own comfortable loudness level during the practice items. This loudness level was then lowered 7.2 dB to mimic the hypophonia (low volume voice) typically found in the speech of persons with Parkinson’s disease [26]. This was done so that electronic amplification did not unnecessarily override the hypophonia of Parkinsonian speech. Once the listening task began, the listeners were not allowed to change the loudness level.

In both phases of the study, speech samples obtained under the different DBS conditions were presented in a random order and listeners were blinded with respect to DBS frequency settings. Intelligibility was assessed by tallying numbers of words correctly transcribed and the associated difficulty ratings by the listeners. Spelling errors were ignored.

### 2.5. Statistical Tests

Statistical analyses included repeated measures analyses of variance (ANOVA), paired t-tests (two-tailed), and Pearson’s correlations (two-tailed). All analyses were performed using SPSS 7.5.

## 3. Results

### 3.1. Intelligibility and Difficulty Ratings

**Figure 1:**
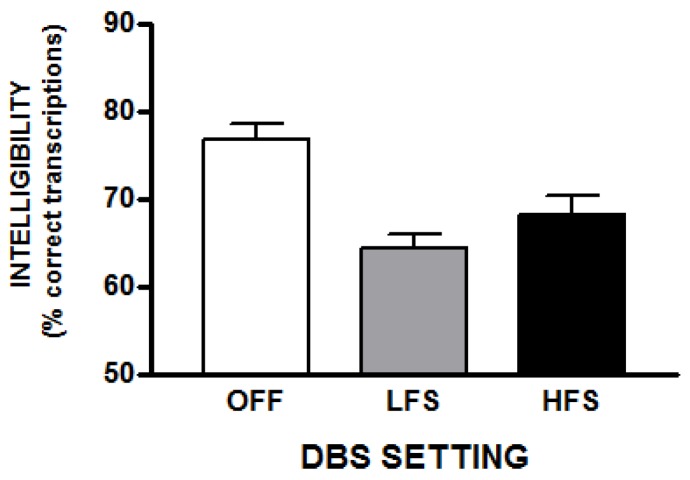
This figure depicts the average intelligibility (% correct transcriptions) with standard errors for the three STN-DBS conditions.

There was a significant effect of DBS setting on the intelligibility measure (percent of words correctly transcribed) [*F*(2,28) = 24.03; *p* < 0.001]. Compared to the DBS off condition, intelligibility was 11% lower with HFS [*t*(14) = −3.63; *p* = 0.003] and 16% lower with LFS [*t*(14) = −8.46; *p* < 0.001]. Intelligibility was significantly higher (approximately 6%) with HFS compared to LFS [*t*(14) = 2.5; *p* = 0.025]. The mean transcription accuracy scores for each stimulation condition are depicted in Figure 1. 

**Figure 2:**
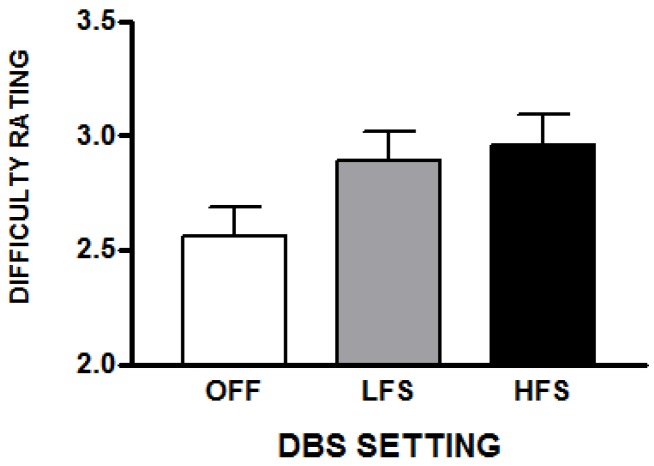
This represents the average difficulty scores (with standard errors) that raters reported while performing transcriptions for the three STN-DBS conditions. The scale was 1 (easy) to 5 (difficult).

Listeners’ ratings of the difficulty experienced in completing the intelligibility task were also affected by the DBS setting [*F*(2,28) = 16.35; *p* < 0.001]. Compared to the DBS off condition, the subjective difficulty was 16% higher for the utterances produced with HFS [*t*(14) = 6.46; *p* < 0.001] and 13% percent higher with LFS [*t*(14) = 4.14; *p* = 0.001]. The difficulty ratings for the two DBS conditions did not differ. These results are depicted in Figure 2.

### 3.2. Relationship Between Average Intelligibility and Average Difficulty Ratings 

The intelligibility scores and difficulty ratings averaged across listeners were negatively correlated in each DBS condition: HFS [*r* = −0.94; *p* = 0.002], LFS [*r* = −0.91; *p* = 0.004], and stimulators off [*r* = −0.9; *p* = 0.005]. The average subjective difficulty experienced while transcribing utterances increased as the average accuracy of the transcriptions decreased regardless of DBS condition. 

There were also relationships with respect to difficulty ratings across DBS conditions. Difficulty ratings with HFS were significantly correlated with those in both the LFS [*r* = 0.731; *p* = 0.002] and off conditions [*r* = 0.865; *p* < 0.001], reflecting difficulty perceiving the speech samples with or without DBS. On the other hand, transcription accuracy in the HFS was correlated with accuracy in LFS [*r* = 0.778; *p* = 0.001], but transcription accuracies in either DBS condition were not correlated with transcription accuracy with DBS off, suggesting that DBS had effects on intelligibility that were not present with DBS off.

### 3.3. Relationship Between Intelligibility and Difficulty Ratings for Each Listener 

The the relationship between individual intelligibility scores and difficulty ratings for each listener was also examined across PD subjects. In contrast to the relationships between intelligibility and difficulty observed when the scores were averaged across listeners, there were no significant correlations between intelligibility and subjective difficulty when the results were examined as individual listeners data averaged across all PD participants. This suggests that listeners may have found some transcriptions difficult but nevertheless intelligible. Furthermore, the factors contributing to the subjective difficulty experienced while transcribing utterances were not uniform across listeners. 

### 3.4. Speech Quality Ratings

Listeners rated the stimuli using five-point scales on four parameters: voice, articulation, fluency, and rate of speech; each scale provided a range from 1 (normal) to 5 (abnormal). These ratings were compared for all three DBS settings, and the possible relationships among these data and intelligibility and difficulty measures were examined.

**Figure 3:**
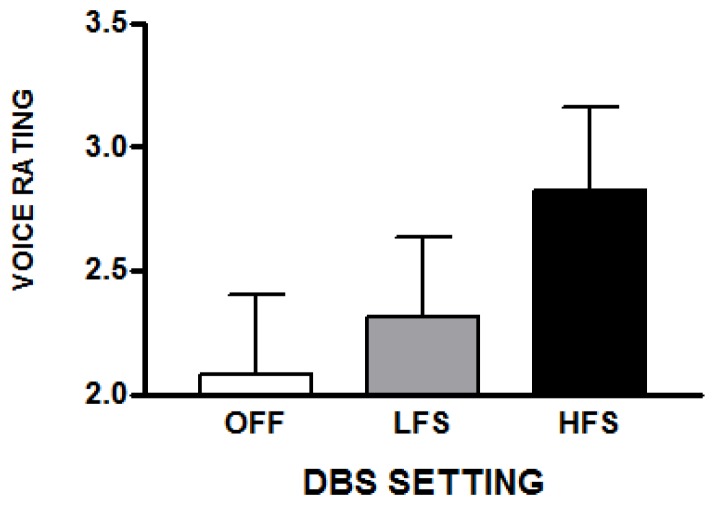
The average subjective ratings of voice abnormalities (with standard errors) that raters reported for a set of spontaneous speech samples obtained during the three STN-DBS conditions. These samples were not used for the transcription task but were used for the voice, articulation, fluency, and rate judgments. The scale was 1 (normal) to 5 (abnormal).

Voice ratings were influenced by the DBS settings [*F*(2,20) = 6.07; *p* = 0.009]. With HFS, voice was rated as more abnormal (36% higher ratings compared to off) [*t*(10) = 3.17; *p* = 0.01], as were LFS ratings (22% higher ratings when compared to off) [*t*(10) = 2.9; *p* = 0.016]. Mean voice ratings did not differ in the HFS and LFS conditions (Figure 3). Similar to the transcription difficulty ratings, voice ratings for HFS utterances were correlated with those produced with LFS [*r* = 0.88; *p* < 0.001] as well as with DBS off [*r* = 0.81; *p* = 0.003]. Voice ratings for LFS were also correlated with voice ratings in the off condition [*r* = 0.72; *p* = 0.01]. DBS appears to have amplified subjective impressions of voice abnormality present without DBS. 

Comparing the voice ratings with intelligibility scores and their associated difficulty ratings, subjective ratings of voice abnormality were negatively correlated with intelligibility [*r* = −0.82; *p* = 0.02] and positively correlated with difficulty [*r* = 0.94; *p* = 0.002] with LFS. Similar but less reliable relationships between rated voice abnormality and intelligibility [*r* = −0.7; *p* = 0.08] and difficulty [*r* = 0.73; *p* = 0.06] were observed with HFS. These relationships were not observed in the DBS off condition. As subjective ratings of voice abnormalities increased, intelligibility accuracy decreased and subjective difficulty increased.

**Figure 4:**
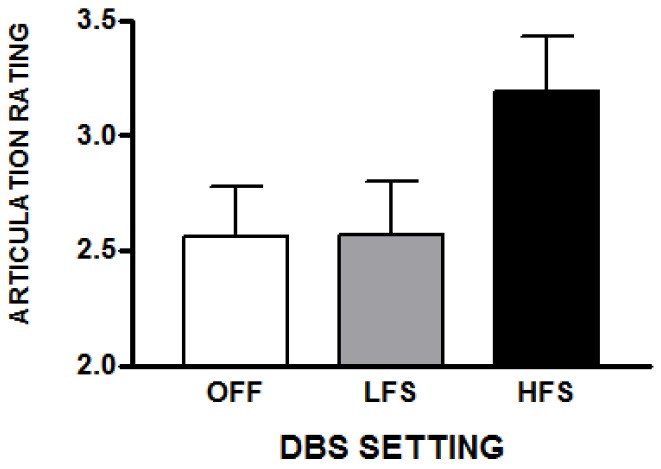
The average subjective ratings of articulation abnormalities (with standard errors) that raters reported for a set of spontaneous speech samples obtained during the three STN-DBS conditions. These samples were not used for the transcription task but were used for the voice, articulation, fluency, and rate judgments. The scale was 1 (normal) to 5 (abnormal).

The DBS settings also affected the subjective ratings of articulation [*F*(2,20) = 4.5; *p* = 0.024]. As with the voice ratings, articulation of HFS utterances was perceived as more abnormal (25% higher) compared to DBS off [*t*(10) = 2.51; *p* = 0.03] as well as compared to LFS (23% higher) [*t*(10) = 2.46; *p* = 0.03]. Articulatory ratings did not differ in the LFS and off conditions (Figure 4). Articulatory ratings in the HFS and LFS conditions were correlated [*r* = 0.74; *p* = 0.01], but neither were correlated with ratings in the off condition, suggesting that DBS introduced articulatory problems not present without DBS. 

Rated articulatory problems were associated with transcription difficulty ratings with DBS off [*r* = 0.9; *p* = 0.006] and with LFS [*r* = 0.9; *p* = 0.01], but not with HFS [*r* = 0.7; *p* = 0.09]. Rated articulatory difficulties were not significantly associated with intelligibility scores in any DBS condition. This indicates that DBS introduced articulation problems that were not uniformly related to intelligibility.

Subjective ratings of fluency or speech rate were not significantly affected by the DBS settings nor were they associated with intelligibility or difficulty rating.

### 3.5. Intelligibility Changes for Individual DBS Subjects

Whereas the previous analyses were performed to characterize average changes in intelligibility effects of DBS frequencies on a group of sophisticated listeners, it is also valuable to examine these effects for each DBS subject. Table 2 presents the percent change in intelligibility scores from the DBS off condition to the LFS and HFS conditions for each individual DBS subject. Two of the subjects showed increased intelligibility in both DBS conditions, two showed decreased intelligibility in both conditions, and three showed mixed results. Table 2 also presents the voice and articulation scores for the off, LFS, and HFS conditions. Higher scores represent greater perceived abnormalities. In parentheses, the percentage changes for each score in the LFS and HFS conditions with reference to the off condition are presented. The columns identified as AVE. (average) represent the average of the percentage in the LFS and HFS conditions. The two subjects with improved intelligibility under both DBS conditions had improved listener ratings (negative change scores) on voice and articulation, with greater improvements on the voice ratings. The two subjects with worsened intelligibility under both DBS conditions had worsened listener ratings (positive change scores) on voice and articulation, The declines in ratings were greater on voice than on articulation. In contrast, for subject 5, intelligibility appeared to be unrelated to either voice or articulation ratings.

## 4. Discussion

These results demonstrate that, in general, both LFS and HFS reduce the intelligibility of spontaneous speech in the range of 11% to 16% compared to the STN-DBS off condition. Reducing the frequency of STN-DBS stimulation did not improve the intelligibility of spontaneous speech. Average intelligibility was significantly higher with HFS compared to LFS. The rated difficulties of the transcription task were not significantly different for LFS and HFS, but they appeared to be slightly higher in the HFS condition. On average, intelligibility was inversely related to the difficulty ratings across DBS conditions. However, when these measures were considered for individual raters, intelligibility and difficulty were not related. The relationships between the difficulty ratings for the transcription task on and off stimulation suggest that the intelligibility difficulties experienced with STN-DBS are also present without stimulation. Individuals who are hard to understand without STN-DBS will be harder to understand with STN-DBS [29].

An examination of the results of individual subjects revealed the complexity of the DBS effects on speech. Two of the DBS subjects had improved intelligibility under both LFS and HFS conditions, while two of the DBS subjects had reduced intelligibility under both LFS and HFS conditions. Subjective voice ratings appeared to be a stronger marker of DBS effects on speech in both situations. In subject 5, intelligibility appeared to be unrelated to the voice and articulation ratings. The contrast between the group results and the individual data illustrate the varied results in the field and serve as a reminder that the DBS effects reflect an interaction between the parameters of the stimulation and the characteristics and condition of the individual with Parkinson’s disease. At this point, there appears to be no general rule regarding DBS parameters and speech effects. 

Consistent with many DBS speech studies, voice was more affected by STN-DBS in the subjective ratings. Voice was rated as most abnormal with HFS, and the rated voice abnormalities in both STN-DBS conditions were correlated with the ratings obtained without STN-DBS. It appears that STN-DBS exacerbates voice problems that occur as a sign of PD. The increased abnormality ratings of voice in both DBS settings occurred despite earlier findings of improved harmonic-to-noise (HNR) ratios in vowels, resulting from DBS [15]. The contrast between subjective ratings and specific acoustic measurements in previous studies reflects the multi-dimensional nature of vowel quality, the effects of task, and the potential interactions among articulation, phonation, and respiratory function.

More abnormal articulatory ratings were also observed with HFS. Rated articulatory abnormalities did not differ between LFS and STN-DBS off. Unlike the voice ratings, the articulatory problems rated in the LFS and HFS conditions were not correlated with the ratings observed in the DBS off condition, suggesting that STN-DBS introduced articulatory problems not present with STN-DBS off. Interestingly, the articulatory abnormality ratings were associated with the difficulty in performing the transcriptions but not with the accuracy of the transcriptions.

The relationships between transcription accuracy and rated difficulty may provide some insight into the variable results found across studies. Generally, the most consistent evidence for negative speech changes with STN-DBS have come from an individual’s subjective impressions recorded using the UPDRS speech rating. The present study demonstrated that while perceived difficulty with comprehending PD speech is associated with reduced intelligibility in general; for individual listeners, this relationship is not consistently present. This suggests that the characteristics of an STN-DBS individual’s spontaneous speech that contribute to difficulties in intelligibility and the speech characteristics that contribute to the subjective sense of difficulty are not the same, and are not uniform across listeners. Furthermore, STN-DBS appears to exacerbate pre-existing voice abnormalities, while introducing new articulation abnormalities. Some characteristics of an STN-DBS individual’s speech may make it difficult to understand but not unintelligible. This distinction may well contribute to the discrepancies in the literature between UPDRS speech ratings and more objective measures, especially when a small number of listeners or raters are used.

Another important factor in considering the STN-DBS speech literature is the fact that most speech assessments involve reading or repetition. Monologue speech has been used in some studies, but even when short monologues are obtained, intelligibility is typically based on read material from standard tests. Unlike standard tests, however, subjects experience and manifest their problems most acutely during spontaneous speech and longer monologues are more likely to better characterize typical spontaneous speech than brief monologues, especially when the quantity of spontaneous speech is reduced. Empirically, the deleterious effects of STN-DBS are more pronounced during spontaneous speech than with reading or repetition [25,26]. 

As we have demonstrated [15,25,26,27], the availability of an external model of the verbal gesture (e.g., reading, repetition) reduces the impact of the problems with phonation and articulation in PD with STN-DBS. It is believed that an external model reduces the burden on the basal ganglia in the production of a complex motor activity [15]. The availability of a verbal model also is likely to reduce problems with planning, which may be affected by STN-DBS. Ahn et al. [30] demonstrated significant changes in the number and position of long pauses during spontaneous speech with STN-DBS. STN-DBS not only differentially affects phonation and articulation at the segmental level, but may well have an effect on planning and sequencing. The support that reading or repetition provides for speech may be analogous to the performance benefits of the availability of external models for limb motor control in individuals with PD [31,32,33].

Consider the phonatory aspect of speech, a component fairly consistently shown to be affected by STN-DBS. Vocal pitch range and variability [16,20], vocal intensity [21,22], and, as mentioned previously, HNR in vowels can be improved with STN-DBS, but the functional impact of these changes may be limited. For example, of 12 acoustic measures, LFS increased fundamental frequency in females but not in males, and intelligibility did not differ when LFS and HFS were compared [22]. In other studies, STN-DBS has resulted in reductions in vocal intensity and vowel duration [23]. Vowel articulation range was found to be reduced with STN-DBS on and off medication, but increased when STN-DBS was accompanied by medication [24].

STN-DBS may have a positive effect on phonation but does not provide a corresponding improvement in articulation accuracy, even for vowels. Sidtis et al. [34] demonstrated normal speakers and those with PD begin sustained phonations with a significantly larger vowel space, which was subsequently reduced at mid-portion of the production. In the same PD subjects, STN-DBS at the clinical frequency eliminated the initial expansion of the vowel space, suggesting a reduction in at least one of the dynamic aspects of vowel articulation. Articulation rate has also been shown to increase with STN-DBS without an improvement in quality [26], but STN-DBS has also been shown to reduce articulation quality [29].

PD gives rise to speech impairments that progress with or without STN-DBS [29,35]; the disease and its treatment affect the multi-system coordination required for normal speech. The interactions among components that include articulatory control, phonation, and respiration are likely to be differentially affected by STN-DBS. It is also the case that speech quality is affected by the extent to which the non-speech signs and symptoms of PD are treated. While there is value in understanding specific effects of STN-DBS, the larger speech production context also reflects individual abilities, the status of PD and its treatment, and the communicative demands placed on the speaker.

## Figures and Tables

**Table 1:** Demographic characteristics of the individuals with DBS who provided speech samples under three different DBS frequency conditions. Males are identified as M, the female as F. Age is in years. HFS frequencies are in Hz and represent the clinical values for each subject. LFS frequency was 60 Hz in all cases. The durations of DBS treatment ranged from two to eight years. The sample size was a function of eligibility criteria (e.g., native language, handedness) and consent for multiple evaluations under different DBS conditions.

PD-DBS								
DBS Subject	Sex	Age	Years of Education	Age at Diagnosis	Years since Diagnosis	Years since DBS	Left HFS	Right HFS
1	M	65	18	45	20	6	185	185
2	M	58	14	44	14	8	185	130
3	M	51	16	34	17	5	185	185
4	F	64	12	48	16	8	130	130
5	M	54	18	38	16	6	185	185
6	M	57	16	44	13	2	185	185
7	M	72	20	59	13	3	185	185
Mean		60.1	14.9	44.6	15.6	5.4		
(SD)		(7.2)	(6.0)	(7.9)	(2.5)	(2.3)		

**Table 2:** The percent change in intelligibility (Intel.) calculated using each subject’s intelligibility using DBS off as the reference value. Positive values represent improved intelligibility, negative values represent reduced intelligibility. Unlike the previous analyses that treated listeners as subjects, this summary averages the intelligibility scores across all listeners for each DBS subject. Two of the DBS subjects (# 1, 2) had increased intelligibility under both LFS and HFS conditions. Two of the DBS subjects (# 3, 4) had decreased intelligibility under both LFS and HFS conditions. The remaining three DBS subjects had mixed results. The table also represents each subject’s voice and articulation (Artic.) ratings by listeners under the DBS Off, LFS, and HFS conditions. The percentage of change with reference to the DBS Off condition is presented in parentheses. The “Rating Ave.” columns represent the average percent changes for the LFS and HFS conditions.

DBS Sub	LFS Intel. Change	HFS Intel.Change	Voice Rating Off	Voice Rating LFS	Voice Rating HFS	Voice Rating AVE.	Artic. Rating Off	Artic. Rating LFS	Artic. Rating HFS	Artic. Rating AVE.
**1**	+11.6	+7.8	3.1	1.6 (−47.3)	2.8 (−10.7)	(−29.0)	2.2	1.3 (−37.4)	2.8 (+30.3)	(−3.6)
**2**	+10.2	+4.2	2.8	2.2 (−22.6)	2.0 (−29.0)	(−25.8)	3.0	2.8 (−6.1)	2.7 (−9.1)	(−7.6)
**3**	−1.5	−39.1	2.0	2.7 (+36.4)	3.5 (+77.3)	(+56.8)	2.2	2.1 (−4.2)	3.5 (+58.3)	(+27.1)
**4**	−6.5	−14.2	1.5	2.5 (+68.7)	3.8 (+162.4)	(+115.6)	2.2	1.7 (−20.9)	4.3 (+95.7)	(+37.4)
**5**	+13.9	−17.5	2.4	3.5 (+50.0)	3.5 (+50.0)	(+50.0)	3.2	3.5 (+8.6)	4.3 (+34.3)	(+21.5)
**6**	+3.5	−16.8	1.2	0.8 (−30.8)	2.0 (−69.2)	(+19.2)	1.9	0.9 (−54.4)	1.5 (−23.8)	(−38.1)
**7**	−5.4	+3.5	1.6	1.6 (0)	1.9 (+16.7)	(+8.3)	1.6	1.9 (+16.7)	2.5 (+50.1)	(+33.4)
